# Increase of *Akkermansia muciniphila* by a Diet Containing Japanese Traditional Medicine Bofutsushosan in a Mouse Model of Non-Alcoholic Fatty Liver Disease

**DOI:** 10.3390/nu12030839

**Published:** 2020-03-20

**Authors:** Mitsue Nishiyama, Nobuhiro Ohtake, Atsushi Kaneko, Naoko Tsuchiya, Sachiko Imamura, Seiichi Iizuka, Shiori Ishizawa, Akinori Nishi, Masahiro Yamamoto, Akinobu Taketomi, Toru Kono

**Affiliations:** 1 Tsumura Kampo Research Laboratories, Tsumura & Co., Ami, Ibaraki 3001192, Japan; oh913685shin@amail.plala (N.O.); kaneko_atsushi@mail.tsumura.co.jp (A.K.); tsuchiya_naoko@mail.tsumura.co.jp (N.T.); imamura_sachiko@mail.tsumura.co.jp (S.I.); iiduka_seiichi@mail.tsumura.co.jp (S.I.); ishizawa_shiori@mail.tsumura.co.jp (S.I.); nishi_akinori@mail.tsumura.co.jp (A.N.); hirokoma@h.email.ne.jp (M.Y.); 2 Department of Gastroenterological Surgery I, Hokkaido University Graduate School of Medicine, Sapporo, Hokkaido 0608638, Japan; taketomi@med.hokudai.ac.jp; 3 Institute of Biomedical Research, Sapporo Higashi Tokushukai Hospital, Sapporo, Hokkaido 0650033, Japan

**Keywords:** Kampo, Bofutsushosan, obesity, NAFLD, *Akkermansia muciniphila*

## Abstract

Non-alcoholic fatty liver disease (NAFLD) is considered a worldwide healthcare problem that mirrors the increased prevalence of obesity. Gut microbiota plays a crucial role in the progression and treatment of NAFLD. Bofutsushosan (BTS), a pharmaceutical-grade Japanese traditional medicine, has long been prescribed in Japan for obesity and obesity-related syndrome. Although BTS has been reported to exert an anti-obesity effect in obese patients as well as various obesity-model animals, its effect on gut microbiota is unknown. Here, the effects of BTS on obesity, liver damage, and the gut microbiome in genetically obese mice, ob/ob, were studied. Seven-week-old ob/ob mice were fed a standard diet with (BTS group) or without (CONT group) 5% BTS for 4 weeks. By comparison to the CONT group, the BTS group showed reduced body weight gain and hyperlipidemia as well as improved liver function. Moreover, gut microbiota in the CONT and BTS group formed a significantly different cluster. Specifically, the genera *Akkermansia*, *Bacteroides* and an unknown genus of the family *Enterobacteriaceae* expanded dramatically in the BTS group. Noteworthy, the population of *Akkermansia muciniphila*, which is reported to elicit an anti-obesity effect and improve various metabolic abnormalities, was markedly increased (93-fold) compared with the CONT group. These results imply that BTS may be a promising agent for treating NAFLD.

## 1. Introduction

Non-alcoholic fatty liver disease (NAFLD) is a progressive fatty liver injury that excludes other causative disorders in patients who do not abuse alcohol. Approximately 25% of NAFLD patients subsequently develop non-alcoholic steatohepatitis (NASH), which increases the risk of developing liver cirrhosis and hepatocellular carcinoma [1,2]. Many studies have demonstrated a strong positive relationship between NAFLD and obesity, and lifestyle modifications are the first-line approach to manage patients with NAFLD [3,4,5,6]. The rapid increase in the prevalence of obesity indicates the importance of environmental effects on pathogenesis and the intestinal tract, which provides the microenvironment.

Recent research suggests the gut microbiota is deeply involved in human health and various disease states including obesity and NAFLD [7,8,9,10]. As a “virtual metabolic and endocrine organ”, gut microbiota influence the health of the host by digesting or metabolizing ingested materials to absorbable, and sometimes biologically active, molecules [11,12]. The gut and liver are connected via the portal vein. As such, the liver is exposed to microbial metabolites such as short-chain fatty acids and secondary bile acids [13]. For example, secondary bile acids, which are metabolized from primary bile acid by gut microbiota, have different affinities for the farnesoid X receptor (FXR). Because FXR is involved in many metabolic processes, the gut microbiota affects host metabolism by affecting secondary bile acids [14,15]. Conversely, bile acids are known to have antibacterial activity [16]. From this perspective, the gut microbiota and liver influence each other. Managing the gut microbiota is now recognized as a potential therapeutic target for obesity and NAFLD [17]. For example, several strains belonging to *Lactobacillus* or *Bifidobacterium* showed anti-obesity effects through species and strain-specific mechanisms [18]. Moreover, *Akkermansia muciniphila* is anticipated to be the next generation of beneficial microbe [19]. Everard et al. reported *A. muciniphila* increases metabolic activity and elicits an anti-obese effect in diet-induced obese (DIO) mice, whereas heat-killed *A. muciniphila* does not [20].

Japanese traditional or “Kampo” medicines are standardized with regard to the quality and quantity of their ingredients and have been approved by the Japanese Ministry of Health and Welfare. Bofutsushosan (BTS), one such Kampo medicine, has long been prescribed in Japan for obesity and obesity-related syndrome. The biological activities of BTS have been demonstrated by clinical studies, including a randomized double-blind placebo-controlled study as well as basic studies [21,22,23]. Ono et al. reported that BTS attenuated development of NASH through induction of adiponectin signaling and phosphorylation of the protein kinase Akt [24]. BTS contains 18 crude drugs, some of which are reported to possess anti-obesity activity (Table S1). Although the mechanisms of action of BTS have been reported in various studies, there is no information regarding its effect on gut microbiota. BTS is indicated for patients with constipation as well as obesity, showing that the gut may be the primary target organ of BTS.

Taken together, these observations suggest that BTS might change the gut microbiota to exert preventive action on the development of NAFLD. The aim of this study was to examine the effect of dietary supplementation of BTS on obesity, liver damage, and the gut microbiome of genetically obese ob/ob mice.

## 2. Materials and Methods

### 2.1. Bofutsushosan (BTS)

BTS was supplied by Tsumura & Co. (Tokyo, Japan) in the form of a powdered extract. The BTS was obtained by spray-drying a hot water extract mixture comprising the following 18 crude components (ratios shown in parentheses): *Angelicae Radix* (1.2), *Paeoniae Radix* (1.2), *Cnidii Rhizoma* (1.2), *Gardeniae Fructus* (1.2), *Forsythiae Fructus* (1.2), *Menthae Herba* (1.2), *Zingiberis Rhizoma* (0.3), *Schizonepetae Spica* (1.2), *Saposhnikoviae Radix* (1.2), *Ephedrae Herba* (1.2), *Rhei Rhizoma* (1.5), *Natrium Sulfricum* (0.7), *Atractylodis Rhizoma* (2.0), *Platycodi Radix* (2.0), *Scutellariae Radix* (2.0), *Glycyrrhizae Radix* (2.0), *Gypsum* (2.0), and *Kasseki* (3.0).

### 2.2. Animals

All experimental procedures were performed according to the Guidelines for the Care and Use of Laboratory Animals of Tsumura & Co. Ethical approval for the experimental procedures used in this study was obtained from the Laboratory Animal Committee of Tsumura & Co (approval no. 06–28, 07–94). Male ob/ob mice (B6.Cg-Lepob/J, six-week-old) and C57BL/6J (six-week-old) were purchased from Charles River Laboratories Japan, Inc. (Kanagawa, Japan). The ob/ob mice are known as leptin-deficient obese mice with the hyperphagia phenotype.

**Figure 1:**
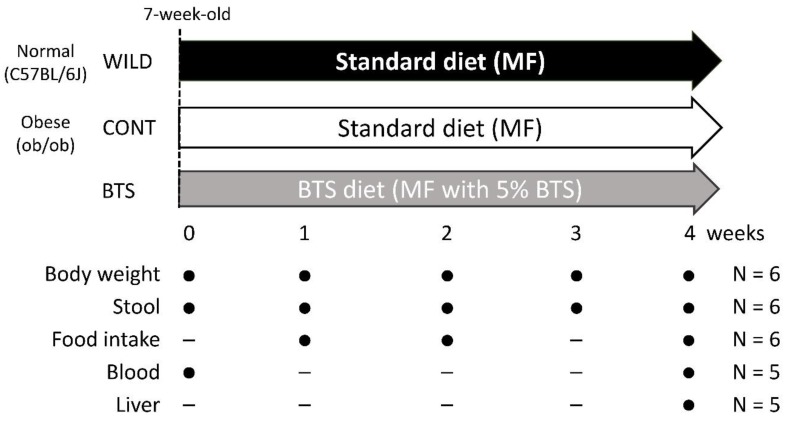
Study design.

After one week of acclimatization (Week 0), male, seven-week-old ob/ob and C57BL/6J mice were randomly divided into groups and fed a standard diet, MF (Oriental Yeast Co., Ltd., Tokyo Japan) or MF supplemented with 5% (w/w) BTS, for four weeks. The dosage of BTS for murine experiments was determined according to previous reports [24]. Body weight was measured weekly (Week 0, 1, 2, 3, 4). Daily food intake was measured at Week 1, 2, and 4. The present study design is as shown in Figure 1.

### 2.3. Histopathological Examination and Blood Biochemistry

At the end of the diet period, all mice were fasted overnight and blood and liver samples obtained under anesthesia. The livers were immediately fixed in 15% buffered formalin. The liver tissues were dehydrated in ethanol and embedded in paraffin according to conventional methods. Sections (45 μm thickness) were stained with hematoxylin and eosin (HE) and examined by light microscopy. Blood samples were centrifuged (1700× *g*, 15 min, 4 °C) and the supernatants collected. Glucose (Glc), total cholesterol (T-Cho), triglyceride (TG), aspartic acid transaminase (AST), and alanine transaminase (ALT) in plasma were measured using an automated biochemical analyzer Toshiba TBA-40FR (Canon Medical Systems Co., Ltd., Tochigi, Japan). The measurement procedures were performed according to the manufacturer’s instructions (FUJIFILM Wako Pure Chemical Corp., Tokyo, Japan).

### 2.4. 16S rRNA Gene Metagenome Sequencing of Stool Samples

During dietary administration, stools were collected weekly and stored at −80 °C until use. DNA was extracted from 10–30 mg stool sample using QIA Amp DNA stool mini kit (Qiagen, Hilden, Germany) according to the manufacturer’s instructions with slight modifications. In brief, stools were mixed with elution buffer supplied as part of the kit and vortexed for 3 min at 1800 rpm using a MicroSmash cell disrupter (Tomy Seiko Co. Ltd., Tokyo, Japan) with zirconia beads. The homogenized samples were centrifuged (10,000× *g* for 30 min) to obtain lysate. Subsequent processing followed the manufacturer’s protocol. DNA concentrations were measured by NanoDrop (LMS. Co. Ltd., Tokyo, Japan). The preparation of 16S rRNA gene metagenome library for MiSeq (Illumina, Inc., San Diego, CA, USA) was performed according to the manufacturer’s protocol. Briefly, 10 ng of DNA template was amplified using Advantage-HF 2 PCR kit (Takara Bio Inc., Shiga, Japan) with universal primers for the 16S rRNA V3–V4 region (forward primer: 5′ TCGTCGGCAGCGTCAGATGTGTATAAGAGACAGCCTACGGGNGGCWGCAG 3′, reverse primer: 5′ GTCTCGTGGGCTCGGAGATGTGTATAAGAGACAGGACTACHVGGGTATCTAATCC 3′). Subsequently, index sequences for each sample were added to both ends of the purified PCR fragments. The concentrations of each amplicon were measured by Quant-iT PicoGreen dsDNA Assay Kit (Thermo Fisher Scientific, Inc., Waltham, MA, USA) and mixed equally. The library was applied to MiSeq Reagent Kit v3 (Illumina, Inc.) and sequence determined using the manufacturer’s standard protocol. Sequence data were processed as follows using the 16S rRNA sequence analysis pipeline, QIIME 1.8.0 [25]. Initially, both sequence reads were joined and sequences with a phred quality score below 20 removed. Chimera elimination by Usearch was performed to remove contaminated sequences. The open reference operational taxonomic unit (OTU) picking was performed against Greengenes 13_8 97% OTU representative sequences. A summary of taxonomy in each sample was obtained using the script “summarize_taxonomy_through_plots.py” in QIIME 1.8.0. The sequences were subsequently deposited to the DDBJ database (BioProject Accession; PRJDB9243).

### 2.5. Quantitative PCR (qPCR)

qPCR for *A. muciniphila* was performed using a specific primer for *A. muciniphila* and universal primer for all bacteria [26]. All primers were purchased from Thermo Fisher Scientific, Inc. as custom primers. DNA extract from stool samples was used as template. Reactions were performed by a standard method using a SYBR Green PCR Kit (Thermo Fisher Scientific, Inc.) and QuantStudio 7 Flex Real-Time PCR system (Thermo Fisher Scientific, Inc.).

### 2.6. Statistical Analysis

Alpha diversity metrics were calculated using the script “alpha_diversity.py” in QIIME 1.8.0. Beta diversity analysis was performed by non-metric multidimensional scaling (NMDS) using Bray–Curtis dissimilarity “metaMDS”. Cluster difference was tested by permutational multivariate analysis of variance (PERMANOVA) using “adonis” in package “vegan” [27] in R 3.5.2 (The R Foundation Conference Committee). The Hierarchical cluster analysis was performed using “hclust” in package “stats” in R 3.5.2. The distance between each variable used Bray–Curtis dissimilarity indices, and the distances between each cluster were obtained by Ward’s method. Univariate analysis between two groups were performed with the Mann–Whitney U test using R 3.5.2.

## 3. Results

### 3.1. Pharmacological Effects of BTS on Obesity, Food Intake, and Hyperlipidemia in ob/ob Mice

**Figure 2:**
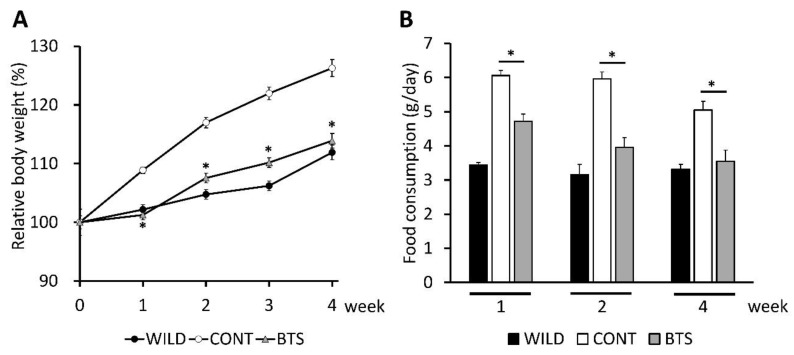
Effects of dietary administration of Bofutsushosan (BTS) on body weight and food intake. Seven-week-old ob/ob and C57BL/6J mice were administered a standard diet with or without 5% (w/w) BTS for 4 weeks. (A) Change of relative body weight based on the value at Week 0. Closed circle, C57BL/6 mice (WILD group); open circle, ob/ob mice fed a standard diet (CONT group); gray triangle, ob/ob mice fed a standard diet supplemented with 5% BTS (BTS group). Data are shown as mean ± SE (*n* = 6). (B) Mice were housed two per cage. Food intake was measured per cage. Closed, open, and gray columns represent WILD, CONT, and BTS groups, respectively. Data are shown as mean ± SE (*n* = 3). *; *p* < 0.05 by Student’s *t*-test with Bonferroni’s correction (CONT vs. BTS).

We first examined time-dependent changes in body weight of leptin-deficient ob/ob mice and wild-type C57BL/6J mice fed a diet supplemented with BTS for 4 weeks. Three groups were studied: CONT group (ob/ob mice fed standard diet), BTS group (ob/ob mice fed standard diet containing 5% BTS), and WILD group (C57BL/6J mice fed standard diet). The body weights at Week 0 (seven-week-old) in the CONT, BTS, and WILD groups were 40.7 ± 2.2, 40.9 ± 1.5, 22.9 ± 0.6 g, respectively. Figure 2A shows the percent of body weight in each week against Week 0. CONT group showed a marked increase in body weight (26.3% ± 6.4%) over the 4 week study period. By contrast, the BTS group gained weight more slowly (13.9% ± 3.7%). The profile obtained for the BTS group was similar to that of the WILD group. Moreover, the BTS group showed no apparent abnormality during the experimental period.

We next evaluated food intake at Week 1, 2, and 4 (Figure 2B). Food intake in the CONT group was more than in the WILD group, as reported earlier [28], whereas that in the BTS group was significantly decreased compared with the CONT group.

Plasma levels of Glc, TG, T-Cho, AST, and ALT were measured to evaluate the effect of BTS on sugar/lipid metabolism and liver injury. We also compared these parameters with seven-week-old mice, i.e., starting age of treatment. As shown in Table 1 (Experiment A), plasma levels of TG, T-Cho, AST, and ALT in ob/ob mice were significantly higher than those of wild-type mice, indicating that metabolic abnormality in ob/ob mice had already occurred by 7 weeks of age. Blood parameters of the CONT and BTS groups at Week 4 showed a significant decrease of T-Cho, AST, and ALT in the BTS group by comparison with the CONT group, whereas Glc and TG levels were not significantly different (Table 1 (Experiment B)).

**Figure 3:**
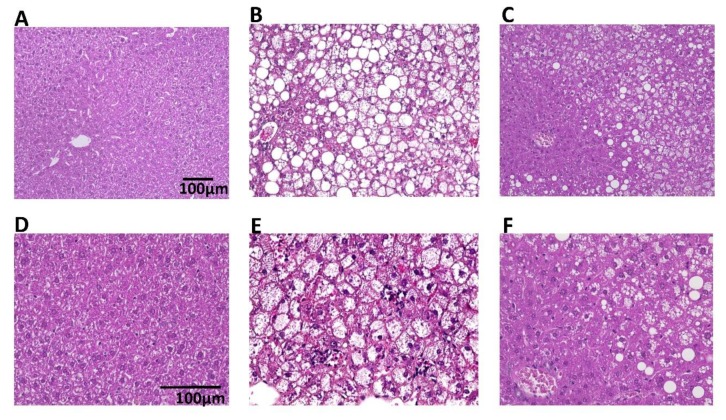
Representative microscopic images of the liver of mice treated with or without BTS. The liver was taken from mice fed with or without 5% (w/w) BTS for 4 weeks. The specimens were stained with hematoxylin and eosin and examined by light microscopy. (**A**,**D**) WILD group, (**B**,**E**) CONT group, (**C**,**F**) BTS group, (**A**–**C**) × 20 magnitude, (**D**–**F**) × 40 magnitude.

An evaluation of hepatic histopathology at Week 4 was performed. The WILD group showed accumulation of glycogen and minimal fat, and few inflammatory cells (Figure 3A,D). Livers from the CONT group showed different sizes of lipid vacuoles, hepatocyte ballooning, and accumulation of inflammatory cells (Figure 3B,E). However, in the BTS group, there was reduced cellular lipid accumulation, hepatocyte ballooning, and accumulation of inflammatory cells compared with the CONT group (Figure 3C,F).

Taken together, BTS suppressed the development of abnormalities in lipid metabolism and liver damage associated with ob/ob mice.

### 3.2. BTS-Dependent Changes of Gut Bacteria, Including those of the Genus Akkermansia

**Figure 4:**
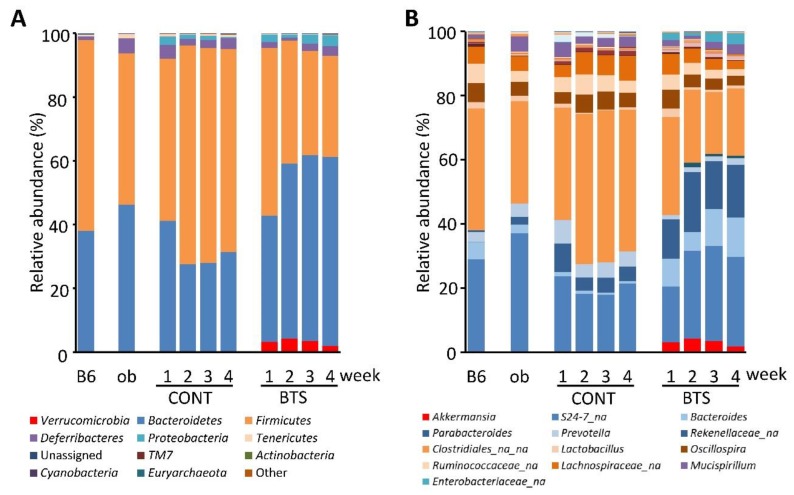
Microbiota composition in stool samples of mice fed a diet with or without BTS. Stools were collected weekly from mice fed with or without 5% (w/w) BTS. Relative abundance of gut microbiota was determined by 16S metagenome sequence analysis. Average relative abundance at each sampling point is shown as bar charts of phylum (**A**) and genus (**B**) levels (*n* = 6). B6, C57BL/6J mice (seven-week-old); ob, ob/ob mice (seven-week-old); CONT, ob/ob mice fed a standard diet; BTS, ob/ob mice fed a standard diet with 5% BTS.

Stool microbiota from ob/ob mice and the wild-type C57BL/6J mice were analyzed by 16S rRNA metagenome sequencing. The number of trimmed, qualified reads in each sample was 6553 ± 2703. Six thousand types of OTU were detected in whole samples (average 575.7 ± 274.0 OTU/sample). The weekly changes of relative abundance at phylum and genus levels of microbiota are shown in Figure 4A,B, respectively. First, we examined the microbiota in ob/ob mice and wild-type mice at 7 weeks of age. No significant difference was found in the relative abundance of phyla exceeding 1%, while several genera showed significant differences between ob/ob mice and the wild-type mice (Table S2).

The shape of microbiota in the BTS group showed a tendency to increase and decrease in the phylum *Bacteroidetes* and *Firmicutes*, respectively. In particular, the phylum *Verrucomicrobia* appeared only in the BTS group throughout the treatment period (Figure 4A). The types of genera were also examined (Figure 4B, Tables S2–S6). Levels of the genus *Bacteroides* and an unknown genus of the family *Enterobacteriaceae* were significantly elevated in the BTS group over the CONT group. By contrast, levels of the genus *Prevotella* were significantly lower in the BTS group compared with the CONT group. Noteworthy, the relative abundance of the genus *Akkermansia* at Week 1 was 3.17% in the BTS group, but below the detection limit (0.001%) for the CONT group (Table S3). Specifically, the genus *Akkermansia* increased the most in the BTS group compared with the CONT group, which continued until Week 3 (Tables S3–S6).

**Figure 5:**
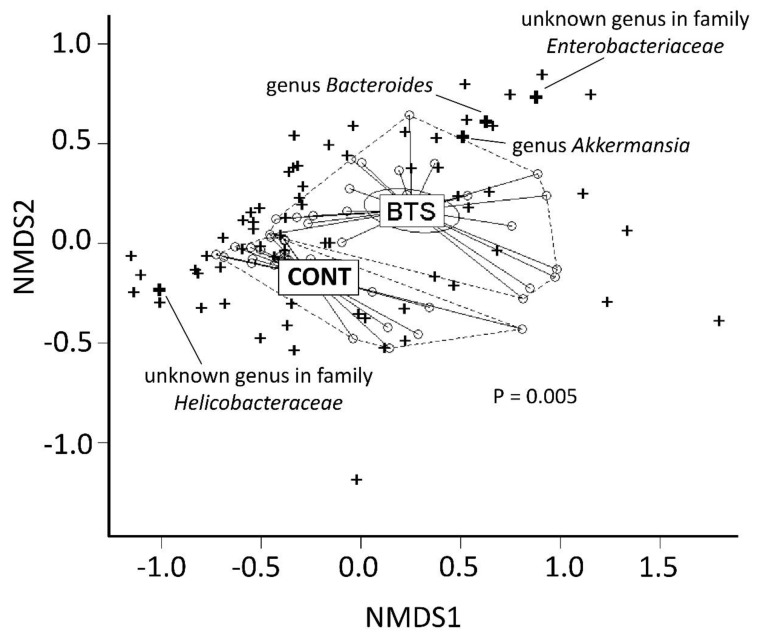
Non-metric multidimensional scaling of stool microbiota in ob/ob mice fed a diet with or without BTS. Similarity of microbiota between CONT and BTS was assessed by non-metric multidimensional scaling (NMDS). Circle, stool sample; Cross, detected genera; Bold cross, genera showing significant differences with 10-fold changes between CONT and BTS groups in Table 2. The limb of each group is shown by a dotted line. The dissimilarity test between CONT and BTS was performed by permutational multivariant analysis of variance test.

The dissimilarity of microbiota in the CONT and BTS groups was visualized using non-metric multidimensional scaling (NMDS). As shown in Figure 5, microbiota in the CONT and BTS groups formed a different cluster, showing a statistical difference (*p* = 0.0050 by PERMANOVA). Because the BTS cluster persisted throughout the treatment period, the Mann–Whitney rank sum test was used (Table 2). In all, 21 bacteria showed a statistical difference between the CONT and BTS groups. In particular, genera *Akkermansia*, *Bacteroides* and an unknown genus of the family *Enterobacteriaceae*, which clustered in the direction of the BTS group (Figure 5), were present at minor levels in the CONT group but expanded dramatically (>1% of relative abundance) following BTS treatment (i.e., 10-fold increase over the CONT group). By contrast, an unknown genus of the family *Helicobacteraceae*, present at 1.24% in the CONT group, was below the detection limit in the BTS group. Among genera whose relative abundance was above 1% in either group, those of the genus *Akkermansia* were most altered in the BTS group (93-fold higher).

### 3.3. Relationship Between Particular Microbes and Body Weight Gain

**Figure 6:**
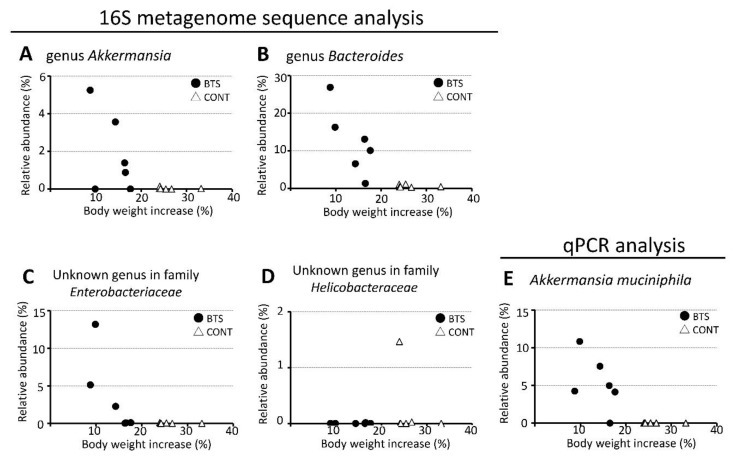
Scattered plot of relative abundance of stool bacteria versus relative increase of body weight. Genera *Akkermansia* (**A**)*, Bacteroides* (**B**), unknown genus in the family *Enterobacteriaceae* (**C**), and unknown genus in the family *Helicobacteraceae* (**D**), were analyzed by 16S metagenome sequencing and plotted versus relative increase of body weight. Only *Akkermansia muciniphila* was analyzed by qPCR (**E**). Open triangle, CONT group; closed circle, BTS group. Increase of body weight (%) was calculated as follows: 100 × ((body weight at Week 4) − (body weight at Week 0))/(body weight at Week 0).

The relationship between microbes in the gut microbiota and body weight gain at Week 4 was studied (Figure 6A–E). The genera *Akkermansia, Bacteroides,* and an unknown genus of the family *Enterobacteriaceae* exerted a negative correlation between relative abundance of bacteria and body weight gain in the BTS group but not in the CONT group. An unknown genus in the family *Helicobacteraceae* was not found to be relevant. Because *Akkermansia muciniphila* was most affected by BTS treatment, the relative abundance of *A. muciniphila* was validated by qPCR (Figure 6E). Akin to the result obtained by 16S metagenomics, the relative abundance of *A. muciniphila* increased only in the BTS group, which correlated with the suppression of body weight gain.

## 4. Discussion

This is the first report to analyze the effect of BTS treatment on the pathophysiology and microbiology of obese model mice (ob/ob). The shape of the gut microbiota in ob/ob mice was rapidly altered in the BTS group, which persisted throughout the treatment period. Major changes to gut microbiota were identified in the BTS group: (i) increase of *A. muciniphila*, genus of *Bacteroides*, and an unknown genus of the family *Enterobacteriaceae*, which were clustered closely in NMDS analysis (Figure 5); (ii) decrease of unknown genus of the family *Helicobacteraceae*.

The rapid increase in the level of *A. muciniphila* was the most interesting finding in this study. Many reports indicate the positive effect of *A. muciniphila* in preventing obesity or metabolic disorders [19,29,30]. Porras et al. reported a negative correlation between the genus *Akkermansia* and NAFLD activity score using DIO mice [31]. Moreover, supplementation of *A. muciniphila* is reported to improve insulin resistance, insulinemia, plasma cholesterol, and AST levels in a double-blind study of overweight and obese patients [32]. Here, supplementation with BTS gave a clear improvement in the plasma levels of total cholesterol, AST, and ALT as well as liver steatosis in ob/ob mice (Table 2, Figure 3). Thus, the present results are in good agreement with previous studies using *A. muciniphila*.

NAFLD is characterized by an abnormal accumulation of fat in the liver related with insulin resistance and can progress into NASH in which steatosis is combined with inflammation. For instance, leak of endotoxin from the intestinal lumen can lead to inflammation in the liver. *A. muciniphila* administration could reverse diet-induced obesity in mice by mediating adipocyte metabolism and gut barrier function [33]. The causative role of *A. muciniphila* in liver disorder in obesity is reviewed comprehensively [34]. *A. muciniphila* modulated the lipid metabolism in circulation, including adipose, liver, and intestine, and the internal metabolite changes caused by *A. muciniphila* were also involved in these actions. Interestingly, *A. muciniphila* is reported to improve expression of epithelial tight junction proteins, occludin and Tjp-1 and suppress lipopolysaccharide (LPS) production by increasing the variety and volume of gut microbes [35]. Everard et al. reported that the mucus layer of diet-induced obesity mice showed 46% thinner compared with that of normal mice, and *A. muciniphila* treatment recovered the thickness, resulting in an anti-obese effect [20]. On the other hand, the thickening activity to the intestinal mucus layer disappeared when they used the heat-killed *A. muciniphila*. These lines indicate that *A. muciniphila* reduces fat accumulation and liver inflammation, which are two essential factors for NASH formation, showing a potential that increase of *A. muciniphila* in gut flora can cause the improvement of NAFLD and/or NASH.

We found a slight negative correlation between dominant microbes that showed >10-fold change by BTS and body weight (Figure 6). The observed reduction of body weight gain by BTS administration cannot be explained by a single microbe such as *A. muciniphila*. Further studies are required to better understand the observed reduction in body weight. For example, a fecal microbiota transplant can be performed to elucidate a direct correlation between the change in microbial population and body weight reduction following BTS treatment. An alternative explanation for reduced body weight gain is a decrease in food intake. Indeed, food intake decreased in the BTS group during the administration period. Thus, BTS may suppress excessive appetite in the present model. Indeed, Azushima et al. reported a suppressive effect of BTS on food intake and concluded this is brought about by modulating the ghrelin system in KKAy mice [36].

This study detected significant increases of genus *Bacteroides* and an unknown genus of the family *Enterobacteriaceae* and a significant decrease of unknown genus of the family *Helicobacteraceae* in the BTS group. Members of the genus *Bacteroides* have been reported to contribute to reinforcement of the intestinal barrier [37], as well as *A. muciniphila*. Multiple Roux-en-Y gastric bypass (RGBY) studies reported a positive correlation with the family *Enterobacteriaceae* and anti-obese activity [38,39]. *Helicobacter pylori*, a member of the family *Helicobacteraceae*, has been reported to be associated with NAFLD [40]. However, the genus *Helicobacter* was not detected in this study. Unfortunately, the biological characteristics of these microbes are not known. Further research into the role of these microbes is required.

The BTS may influence the shape of microbiota in several ways. The antibacterial activity of BTS is one possible mechanism. Among BTS components, *Menthae Herba*, *Zingiberis Rhizoma*, *Rhei Rhizoma, Paeoniae Radix*, *Atractylodis Rhizoma*, and Forsythia Fruit are reported to possess antibacterial activity [41,42,43,44,45,46,47,48]. Here, the total number of bacteria was estimated by qPCR to assess the possible influence of BTS on bacterial count. No significant difference in copy number of total bacteria between the CONT and BTS group was observed (Figure S1). Prebiotic-like effects were also considered. Fibers, polysaccharides, and polyphenols are contained in plant materials and some of them are reported to influence the microbiota [49]. Furthermore, many active compounds, including flavonoids, are consumed as glycosides that need to be deglycosylated by specific gut microbes to display biological activity. Several reports suggest a relationship between flavonoids and the genus *Akkermansia* [50].

Component crude drugs of BTS, *Atractylodis Rhizoma* and *Rhei Rhizoma*, which are reported to augment the genus *Akkermansia* in the gut [51,52], are known to include bioactive glycosides atractylodis and sennoside, respectively. Chen et al. reported that the genus *Akkermansia* was increased by administration of another Kampo drug, orengedokuto, in a high-fat diet and streptozotocin induced type 2 diabetic model rat [53]. *Gardeniae Fructus* and *Scutellariae Radix* are common crude drugs in both orengedokuto and BTS. Specifically, *Gardeniae Fructus* contains geniposide, a glycoside of genipin. *Scutellariae Radix* contains various types of flavonoid glycoside such as baicalin, wogonoside, oroxylin A-7-*O*-glucronide, liquiritin, and isoliquiritin. Therefore, it is possible that these glycosides in BTS alter gut microbiota including the genus *Akkermansia* via a prebiotic mechanism during BTS administration. For example, geniposide has been reported to be effective for liver protection and must be metabolized by gut microbiota for its activation [54]. Baicalin has been reported to possess anti-obese and liver steatosis suppression activities [55]. 1,2,3,4,6-penta-*O*-galloyl-β-D-glucose, a polyphenolic compound highly enriched in *Paeoniae Radix*, has also been reported to have anti-diabetic activity and is metabolized by gut microbiota. Furthermore, gallotannin is reported to show prebiotic effects on Bifidobacteria and lactic acid bacteria [56]. Thus, if specific microbes contribute to the metabolism of these glycosides, BTS might act as a prebiotic-like agent.

It is also important to consider mechanisms by which BTS might exert its effects via action on the liver and/or surrounding organs. Indeed, crude drugs in BTS are reported to have anti-obese activity (Table S1). Moreover, ingredients in BTS have been shown to elicit microbe-independent activities against obese or metabolic disorders. For example, baicalein, one of the main active compounds in *Scutellariae Radix*, significantly improved hyperglycemia, glucose tolerance, and blood insulin levels in obese diabetic mice by directly modulating pancreatic β-cell function [57]. BTS may suppress obesity and/or metabolic disorder, including NAFLD, in ob/ob mice via microbiota-independent and/or -dependent activities.

In order to advance our detailed research, study needs to be conducted using ingredients of BTS. In general, it is difficult to find out bioactive compounds from natural products and to obtain natural compounds sufficient for evaluating their activity. However, there are many trials to identify active compounds by unique and advanced technology. Farzaneh et al. demonstrated the comprehensive screening systems to research bioactive compound in medicinal plants [58]. Interestingly, they have reported novel modeling in which extraction efficiency and biological activity of target compounds could be increased by microwave irradiation or ultrasound treatment [59,60,61,62]. Recently, there has been a universal propensity to application of natural phytochemicals because of existence of substituents with bioactive potentials, well-being advantages, and functional ingredients [63]. It is a future consideration to uncover active compounds in BTS to understand the pharmacological mechanism of BTS and to develop effective drugs for obesity and obesity-related syndrome.

Our study, which reveals the beneficial effects of BTS on obesity and liver damage, is consistent with earlier reports [24,64]. These findings confirm the effectiveness of BTS on obesity and NAFLD regardless of differences in animals and diet.

The limitations of this study are as follows: (1) bioactive compound in BTS was not identified; (2) the relationship between gut microbe alteration and body weight gain was not evaluated statistically; (3) the study was not designed to clarify whether the effects of BTS on appetite, body weight gain, and liver damage were due to BTS-associated alteration of gut microbiota; (4) it was unclear whether the changes in the microbiota are primarily driven by BTS or whether BTS led to reduced appetite and the microbiota changes were due to the decreased food intake. Thus, conclusions based on these exploratory results should be made with caution. Further studies that address these limitations are necessary in the future.

In conclusion, we have verified that BTS has a beneficial effect on obese and obese-induced liver injury in ob/ob mice. The beneficial effects of BTS in the treatment of NAFLD are associated with changes in gut microbiota, in particular *A. muciniphila*.

## Figures and Tables

**Table 1:** Plasma metabolic parameters.

	Glc (mg/dL)	T-Cho (mg/dL)	TG (mg/dL)	AST (IU/L)	ALT (IU/L)
Experiment A.					
C57BL/6J	119.3 ± 10.0	47.0 ± 1.4	29.0 ± 11.0	18.8 ± 2.9	11.3 ± 1.9
ob/ob	198.3 ± 45.9	104.5 ± 18.2 *	73.3 ± 14.2 *	110.3 ± 24.6 *	126.8 ± 26.9 *
Experiment B					
CONT group	145.3 ± 24.3	107.5 ± 9.9	19.8 ± 2.8	163.0 ± 29.7	167.0 ± 25.9
BTS group	118.0 ± 41.8	86.8 ± 6.0 *	17.0 ± 1.6	65.2 ± 25.8 *	55.2 ± 24.0 *

Experiment A, Plasma metabolic parameters in C57BL/6J and ob/ob mice (seven-week-old) fed a standard diet; Experiment B, Plasma metabolic parameters in ob/ob mice with or without BTS administration for 4 weeks. The plasma samples were collected after overnight fasting and metabolic parameters measured. Data are shown as average ± SD (*n* = 5). Glc; glucose, T-Cho; total cholesterol, TG; triglyceride, AST; Aspartate transaminase, ALT; Alanine transaminase, *: *p* < 0.05 by Student’s *t*-test.

**Table 2:** Relative abundance of microbiota (genus level) throughout administration of 5% BTS in ob/ob mice.

Phylum	Genus	CONT Group (%) Mean ± SD	BTS Group (%) Mean ± SD	Ratio	*p*-Value
*Verrucomicrobia*	*Akkermansia*	0.03 ± 0.07	**3.14 ± 2.25**	**93.36**	1.60 × 10^−6^
*Bacteroidetes*	*Bacteroides*	0.91 ± 0.85	**9.58 ± 6.90**	**10.50**	1.69 × 10^−11^
	*Prevotella*	**5.24 ± 3.14**	**1.60 ± 0.97**	0.30	3.32 × 10^−9^
	*Parabacteroides*	**5.58 ± 5.79**	**15.53 ± 6.61**	2.78	3.83 × 10^−8^
	*[Prevotella]*	BLD	0.78 ± 1.13	777.97	2.31 × 10^−4^
	unknown genus in order *Bacteroidales*	BLD	0.01 ± 0.01	5.94	2.07 × 10^−2^
*Firmicutes*	unknown genus in family *Erysipelotrichaceae*	0.14 ± 0.13	0.84 ± 0.55	6.00	1.77 × 10^−9^
	unknown genus in order *Clostridiales*	**43.33 ± 11.29**	**23.36 ± 15.45**	0.54	2.87 × 10^−6^
	*[Eubacterium]*	<0.01	0.11 ± 0.14	167.26	1.35 × 10^−5^
	*Anaerotruncus*	BLD	0.01 ± 0.01	9.82	5.19 × 10^−4^
	unknown genus in family *Christensenellaceae*	0.01 ± 0.01	0.02 ± 0.03	4.07	1.06 × 10^−2^
	*Clostridium*	0.01 ± 0.01	<0.01	0.39	1.41 × 10^−2^
	unknown genus in family *Lachnospiraceae*	0.14 ± 0.28	0.05 ± 0.08	0.37	2.38 × 10^−2^
	*Streptococcus*	<0.01	0.01 ± 0.01	6.29	2.44 × 10^−2^
	*Coprobacillus*	<0.01	0.03 ± 0.04	6.40	3.58 × 10^−2^
	*Sporosarcina*	0.01 ± 0.03	BLD	0.12	4.10 × 10^−2^
	*Ruminococcus*	0.98 ± 0.70	0.65 ± 0.52	0.66	4.28 × 10^−2^
*Proteobacteria*	unknown genus in family *Enterobacteriaceae*	0.06 ± 0.27	**2.39 ± 3.55**	**37.59**	2.03 × 10^−8^
	unknown genus in family *Helicobacteraceae*	**1.24 ± 2.22**	<0.01	<**0.01**	8.06 × 10^−3^
*Tenericutes*	unknown order in *RF39*	0.23 ± 0.26	0.07 ± 0.05	0.30	2.52 × 10^−4^
*Actinobacteria*	*Bifidobacterium*	BLD	0.03 ± 0.06	30.56	2.42 × 10^−3^

Relative abundance of microbes throughout administration with 5% (w/w) BTS determined at the genus level. Statistical differences between CONT and BTS groups were examined by the Mann–Whitney rank sum test, showing genus with significant alteration in *p*-value < 0.05. When genus could not be detected, abundance value was provisionally assigned as 0.001%. Ratio was calculated as BTS group/CONT group. CONT, ob/ob mice fed a standard diet. BTS, ob/ob mice fed a standard diet with 5% BTS. BLD, below limit of detection in all samples; bold, relative abundance >1%; underlined bold, ratio was >10 or <0.1 and the relative abundance >1%.

## Supplementary Materials

The following are available online at https://www.mdpi.com/2072-6643/12/3/839/s1, Table S1: Components of Bofutsushosan and their reported biological activities, Table S2: Relative abundance of microbiota (genus level) in mice (7-week-old, no treatment), Table S3: Relative abundance of microbiota (genus level) in ob/ob mice (one week on dietary administration of 5% BTS), Table S4: Relative abundance of microbiota (genus level) in ob/ob mice (two weeks on dietary administration of 5% BTS), Table S5: Relative abundance of microbiota (genus level) in ob/ob mice (three weeks on dietary administration of 5% BTS), Table S6: Relative abundance of microbiota (genus level) in ob/ob mice (four weeks on dietary administration of 5% BTS), Figure S1: Copy number of 16S rRNA gene in stool samples.
