# Supplementary material for: Increase of Akkermansia muciniphila by a Diet Containing Japanese Traditional Medicine Bofutsushosan in a Mouse Model of Non-Alcoholic Fatty Liver Disease
Source: Nutrients. 2020 Mar 20;12(3):839. doi: 10.3390/nu12030839 (PMC7146306; doi:10.3390/nu12030839)
Supplement: Supplementary file 1 [file nutrients-12-00839-s001.pdf]

Table S1. Components of Bofutsushosan and their reported biological activities.

Crude drug	Reported biological activity	ref
Angelicae Radix	Anti-obesity in HFD mice	65
Paeoniae Radix	Anti-obesity and effect on microbiota in HFD mice	66
Cnidii Rhizoma	None	
Gardeniae Fructus	Anti-hyperglycemia and anti-obesity in STZ diabetic mice	67
Forsythiae Fructus	None	
Menthae Herba	None	
Zingiberis Rhizoma	GLUT5 inhibition<br>Cholesterol uptake inhibition into Caco-2 cells	68<br>69
Schizonepetae Spica	None	
Saposhnikoviae Radix	GLUT5 inhibition	66
Ephedrae Herba	Anti-obesity	70
Rhei Rhizoma	Suppression of differentiation of 3T3-L1 cells	71
Natrium Sulfricum	None	
Atractylodis Rhizoma	Anti-obesity in HFD rat with LPS	51
Platycodi Radix	Anti-obesity by activating AMPK in db/db mice	72
Scutellariae Radix	Anti-obesity in db/db mice	73
Glycyrrhizae Radix	None	
Gypsum	None	
Kasseki (mainly composed of aluminium silicate hydrate and silicon dioxide)	None	

ref, references

Table S2. Relative abundance of microbiota (genus level) in mice (7 week-old, no treatment).

Phylum	Genus	C57BL/6J (%) Mean ± SD.	ob/ob (%) Mean ± SD	Ratio	*p*-value
Verrucomicrobia	*Akkermansia*	0.04 ± 0.04	<0.001	0.08	0.040
Unassigned	Unassigned	0.31 ± 0.09	0.10 ± 0.04	0.33	0.002
Bacteroidetes	*Parabacteroides*	0.11 ± 0.10	**2.37 ± 1.76**	**21.73**	0.002
	unknown genus in family *Rikenellaceae*	0.56 ± 1.06	<0.001	0.00	0.017
Cyanobacteria	unknown genus in order *Streptophyta*	BLD	0.01 ± 0.01	[illegible]1.27	0.028
Firmicutes	*Turicibacter*	0.10 ± 0.06	BLD	0.00	0.003
	unknown genus in order *Clostridiales*	0.48 ± 0.37	0.04 ± 0.08	0.09	0.008
	unknown genus in family *Clostridiaceae*	0.08 ± 0.03	BLD	0.00	0.003
	Coprococcus	0.84 ± 0.36	0.13 ± 0.14	0.15	0.008
	Dorea	0.14 ± 0.08	0.03 ± 0.05	0.20	0.029
	[Ruminococcus]	0.24 ± 0.09	0.10 ± 0.09	0.44	0.045
	unknown genus in family *Erysipelotrichaceae*	0.04 ± 0.02	0.15 ± 0.08	3.94	0.015

Genera that showed a significant difference between C57B/6J and ob/ob mice by Mann-Whitney *U* (MWU) test ($p < 0.05$, $n = 6$) are listed. When genus could not be detected, abundance value was provisionally assigned as 0.001%. Ratio was calculated as ob/ob group / C57BL/6J group. BLD, below limit of detection in all samples; bold, relative abundance >1%; underlined bold, ratio was >10 or <0.1 and relative abundance was >1%.

Table S3. Relative abundance of microbiota (genus level) in ob/ob mice (one week on dietary administration of 5% BTS).

Phylum	Genus	CONT group (%) Mean ± SD	BTS group (%) Mean ± SD	Ratio	*p*-value
Verrucomicrobia	*Akkermansia*	BLD	**3.17 ± 1.59**	**3165.07**	0.003
Bacteroidetes	*Bacteroides*	**1.30 ± 0.77**	**8.56 ± 7.71**	6.57	0.002
	Prevotella	**7.33 ± 4.64**	**1.28 ± 0.81**	0.18	0.009
Firmicutes	*Coprococcus*	0.08 ± 0.09	0.58 ± 0.32	7.04	0.008
	[Ruminococcus]	0.10 ± 0.12	0.38 ± 0.21	3.86	0.012
	Anaerotruncus	BLD	0.02 ± 0.02	20.16	0.010
	unknown genus in family *[Mogibacteriaceae]*	0.01 ± 0.02	0.06 ± 0.05	4.16	0.042
Proteobacteria	*Unknown genus of family Enterobacteriaceae*	0.22 ± 0.53	**2.35 ± 3.39**	**10.81**	0.025
Tenericutes	*Unknown genus of order RF39*	0.43 ± 0.47	0.04 ± 0.04	0.10	0.026

Bacterial alteration by BTS administration for 1 week was investigated. Genera that showed significant difference between CONT and BTS groups by Mann-Whitney *U* (MWU) test ($p < 0.05$, $n = 6$) are listed. When genus could not be detected, abundance value was provisionally assigned as 0.001%. Ratio was calculated as BTS group / CONT group. BLD, below limit of detection in all samples; bold, relative abundance >1%; underlined bold, ratio was >10 or <0.1 and relative abundance was >1%.

Table S4. Relative abundance of microbiota (genus level) in ob/ob mice (two weeks on dietary administration of 5% BTS).

Phylum	Genus	CONT group (%) Mean ± SD	BTS group (%) Mean ± SD	Ratio	*p*-value
Verrucomicrobia	*Akkermansia*	0.01 ± 0.02	**4.16 ± 2.40**	**<u>277.51</u>**	0.019
Bacteroidetes	*Bacteroides*	**1.06 ± 1.45**	**5.87 ± 4.04**	5.56	0.009
	Parabacteroides	**4.09 ± 2.23**	**18.64 ± 9.60**	4.56	0.002
	Prevotella	**4.21 ± 2.23**	**1.55 ± 0.63**	0.37	0.015
Firmicutes	unknown genus in order *Clostridiales*	**46.68 ± 9.42**	**22.76 ± 15.37**	0.49	0.015
	unknown genus in family *Erysipelotrichaceae*	0.10 ± 0.08	**1.07 ± 0.83**	**<u>10.87</u>**	0.009
Proteobacteria	unknown genus in family *Enterobacteriaceae*	BLD	0.92 ± 1.15	919.86	0.003

Bacterial alteration by BTS administration for 2 weeks was investigated. See method in Table S3.

Table S5. Relative abundance of microbiota (genus level) in ob/ob mice (three weeks on dietary administration of 5% BTS).

Phylum	Genus	CONT group (%) Mean ± SD	BTS group (%) Mean ± SD	Ratio	*p*-value
Verrucomicrobia	*Akkermansia*	0.08 ± 0.12	**3.41 ± 2.68**	**<u>40.51</u>**	0.034
Bacteroidetes	*Bacteroides*	0.66 ± 0.25	**11.50 ± 5.83**	**<u>17.32</u>**	0.002
	Parabacteroides	**4.65 ± 2.42**	**14.86 ± 4.81**	3.20	0.004
	Prevotella	**4.86 ± 1.82**	**1.50 ± 0.43**	0.31	0.002
Firmicutes	unknown genus in order *Clostridiales*	**47.35 ± 4.14**	**19.28 ± 11.92**	0.41	0.002
	unknown genus in family *Erysipelotrichaceae*	0.11 ± 0.09	0.85 ± 0.38	8.03	0.002
	[Eubacterium]	<0.001	0.20 ± 0.21	204.38	0.010
Proteobacteria	unknown genus in family *Enterobacteriaceae*	0.01 ± 0.02	**2.82 ± 3.79**	**<u>277.50</u>**	0.004

Bacterial alteration by BTS administration for 3 weeks was investigated. See method in Table S3.

Table S6. Relative abundance of microbiota (genus level) in ob/ob mice (four weeks on dietary administration of 5% BTS).

Phylum	Genus	CONT group (%) Mean ± SD	BTS group (%) Mean ± SD	Ratio	*p*-value
Actinobacteria	*Bifidobacterium*	<0.001	0.10 ± 0.10	98.29	0.010
Bacteroidetes	*Bacteroides*	0.63 ± 0.38	**12.38 ± 8.81**	**19.72**	0.002
	Parabacteroides	**4.68 ± 3.09**	**16.31 ± 6.38**	3.48	0.015
	Prevotella	**4.59 ± 2.89**	**2.05 ± 1.65**	0.45	0.041
	[Prevotella]	<0.001	0.79 ± 0.84	789.80	0.028
Firmicutes	unknown genus in family *Erysipelotrichaceae (2)*	0.10 ± 0.10	0.90 ± 0.56	8.84	0.004
	[Eubacterium]	<0.001	0.09 ± 0.07	90.73	0.010
Proteobacteria	unknown genus in family *Enterobacteriaceae*	0.03 ± 0.04	**3.46 ± 5.17**	**129.49**	0.028

Bacterial alteration by BTS administration for 4 weeks was investigated. See method in Table S3.

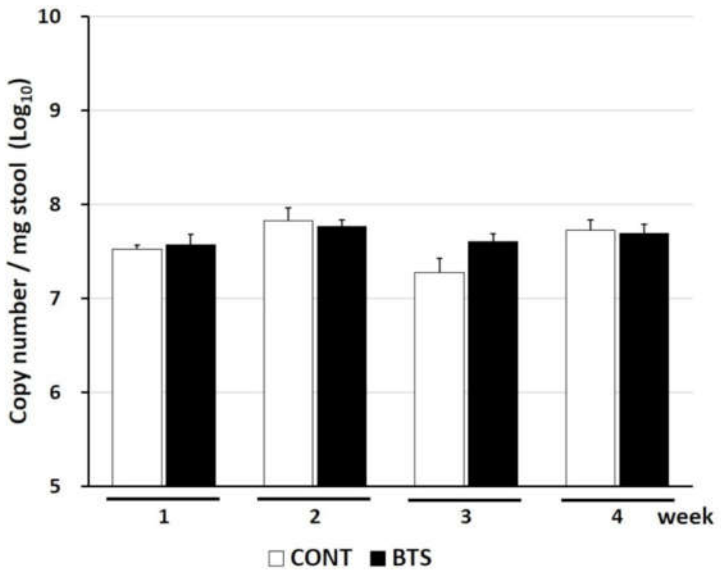

Figure S1. Copy number of 16S rRNA gene in stool samples.

DNA in stool samples was extracted and the copy number of rRNA gene was quantified by qPCR. The Universal primer used to detect all bacteria is described in Materials and Methods. Data are shown as mean ± SE (n = 6).
